# Altered responses of end‐expiratory lung volume and upper airway patency to body posture in diet‐induced obese mice

**DOI:** 10.14814/phy2.15072

**Published:** 2021-10-21

**Authors:** Tatsunori Takahashi, Noriaki Sakai, Seiji Nishino

**Affiliations:** ^1^ Department of Medicine Jacobi Medical Center Albert Einstein College of Medicine Bronx New York USA; ^2^ Sleep and Circadian Neurobiology Laboratory Department of Psychiatry and Behavioral Sciences Stanford University School of Medicine Palo Alto California USA

**Keywords:** body fat distribution, body position, computed tomography, obesity, physiology

## Abstract

**Objective:**

Although both obesity and body posture are important factors affecting end‐expiratory lung volume (EELV) and upper airway patency, the influence of those factors on EELV and the association between EELV and upper airway calibers are still unknown in mice. This study examined such interaction effects in obese mice to test the hypothesis that obese mice have decreased EELV accompanied by structural alterations of the upper airway.

**Methods:**

A high‐resolution in vivo micro‐computed tomography was utilized to scan anesthetized lean and diet‐induced obese mice in the prone and supine positions, followed by quantifying lung volume and analyzing upper airway morphology.

**Results:**

There was a statistically significant interaction between the effects of body weight and posture on both EELV (*p* = 0.0049, *η* ^2^ = 0.1041) and upper airway calibers (*p* = 0.0215, *η* ^2^ = 0.6304). In lean mice, EELV in the prone position was significantly larger than in the supine position (prone EELV = 193.22 ± 9.10 µl vs. supine EELV = 176.01 ± 10.91 µl; *p* = 0.0072), whereas obese mice did not have such an improvement in EELV in the prone position (prone EELV = 174.37 ± 20.23 µl vs. supine EELV = 183.39 ± 17.49 µl; *p* = 0.0981) and tended to have a smaller upper airway when EELV was low based on Spearman's correlation analysis.

**Conclusions:**

These data indicate that obesity is an important factor compromising both EELV and upper airway calibers in a posture‐dependent manner even in mice, which should be taken into consideration in future studies regarding upper airway collapse and lung mechanical properties using mice.

## Graphical abstract

This study examined effects of obesity and body posture on end‐expiratory lung volume and upper airway calibers in mice. The reduced chest wall compliance secondary to obesity diminished the advantageous effect of the prone position on lung mechanical properties. Obese mice had a smaller upper airway compared to lean mice when EELV was low.

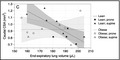

## 1 INTRODUCTION

The influence of end‐expiratory lung volume (EELV) on upper airway dimensions and collapsibility is well recognized in obese subjects with obstructive sleep apnea (Hoffstein et al., 1984; Stadler et al., 2009). Intra‐abdominal fat causes the cephalic displacement of the diaphragm, resulting in decreased EELV (Stadler et al., 2009). Additionally, chest wall fat affects EELV and lung compliance by compressing the chest wall inward (Babb et al., 2008). The resultant decrease in EELV reduces intraluminal pressure and axial tension of the upper airway and, in turn, compromises upper airway patency (Stadler et al., 2009).

Although obese mouse models have been widely used for understanding the underlying mechanisms of upper airway collapse, the role of EELV in the pathogenesis of increased upper airway collapsibility still remains to be elucidated in mice due to technical difficulties measuring EELV (Irvin & Bates, 2003). The fact that the New Zealand obese mouse occasionally sleeps upright suggests that both obesity and posture likely alter lung mechanical properties including EELV even in mice (Brennick et al., 2009).

In order to test the hypothesis that obese mice have decreased EELV accompanied by structural alterations of the upper airway, we examined the interaction effects of body weight and posture on EELV. In this study, we utilized a high‐resolution micro‐computed tomography (µCT) to scan lean and obese mice in the prone and supine positions.

## 2 METHODS

### 2.1 Ethical approval

All procedures were approved by the Committee on the Ethics of Animal Experiments of the Stanford University Administrative Panel on Laboratory Animal Care (protocol no. 21646) and complied with the USDA Animal Welfare Act.

### 2.2 Animal experiments

Ten male C57BL/6J mice at 7 weeks of age were obtained from The Jackson Laboratory. Five control mice were fed a regular diet (3.1 kcal/g, 6.2% fat, 18% kcal from fat. 2918, Envigo) while five experimental mice were maintained with a high‐fat diet (5.21 kcal/g, 34.9% fat, 60% kcal from fat. D12492, Research Diets) for 13 weeks. Food and water were provided ad libitum. After µCT imaging, mice were euthanized with carbon dioxide in compliance with an approved protocol.

### 2.3 4D time‐resolved μCT imaging

To date, the upper airway of mice has been evaluated in two different body positions: prone and supine (Brennick et al., 2009; Polotsky et al., 2011, 2012). In particular, morphological evaluations were conducted in the supine position using magnetic resonance imaging although the influence of EELV on the upper airway was not studied in the previous study (Brennick et al., 2009). Since the goal of this present study is to clarify the interaction effects of body weight and posture on EELV, we scanned lean and obese mice placed in the physiological position (i.e. prone) and the most common experimental position (i.e. supine) using an in vivo µCT scanner (SkyScan 1276, Bruker). Each mouse was free breathing under isoflurane anesthesia with a target respiratory rate of approximately 60–70 breaths per minute. A custom bed and mask made of polystyrene foam were used to maintain natural neck alignment. Continuous heated airflow was provided to prevent temperature loss during anesthesia. The scanning mode was set 360 degrees, step and shoot scanning without average framing. We applied voltage 70 kV and a 0.5‐mm aluminum filter, with the scan image voxel size of 70 µm and a binning of 500, which is the lowest binning available in SkyScan 1276. A high binning mode such as 2,000 and 4,000 provides higher resolution images but created blurry images of the pharynx due to a long camera exposure time per frame (e.g. >350 ms), resulting in the fusion of inspiratory and expiratory images. On the other hand, the above setting allowed a very short exposure time per frame of 44 ms with a sufficient field of view and resolution to analyze both the upper airway and lung. Twenty images were acquired per rotation step of 0.8 degrees to ensure at least one cycle of breath was scanned at every step. The duration of each scan was approximately 11 minutes.

To allow time‐based retrospective synchronization (De Langhe et al., 2012), the precise time of each breathing signal was recorded in real time by video‐recording the respiratory movement of a small, white polystyrene foam block fixed to the mouse's chest wall for better visualizing the breathing movement in the dark background. A physiological monitoring software (SkyScanVisual, Bruker) converted the recorded breathing movement into a waveform signal for retrospective synchronization. We divided each breathing cycle into eight equal‐interval phases and sorted all the projection images into the eight corresponding bins using Tsort (Bruker) to obtain two image bins corresponding to the peak inspiration and end‐expiration phases, respectively. After each scan and sorting, the projection images were reconstructed using NRecon (Bruker), followed by converting the set of reconstructed slices to DICOM files using DICOM converter (Bruker).

### 2.4 Quantification of lung volume and adipose tissue amount

CTAn (Bruker) was used to quantify EELV and end‐inspiratory lung volume (EILV). The 2D images showing the most cranial or caudal edge of the lung were set as the top and bottom of the volume of interest (VOI), respectively. We manually created a separate VOI that contained either the right or left lung and extracted an intensity histogram from each lung. The extracted histograms showed a unimodal distribution. The Hounsfield unit value representing the peak in the histogram is the density of the boundary between air and the alveolar epithelium.

CTAn (Bruker) was used to quantify EELV and EILV by separating the aerated lung from the tissue lung with a mouse‐specific threshold that was obtained from the intensity histogram of the lung (Bell et al., 2018). Thus, the peak Hounsfield unit values from each lung were averaged and used as a mouse‐specific threshold to separate the aerated lung from the tissue lung. After determining the threshold, another VOI containing the thoracic cavity was created. The thresholding plug‐in was run with the threshold in the Custom Processing tab of CTAn to generate the aerated lung. We refined images by removing all structures outside the lung with the sweeping feature of the despeckle plug‐in, followed by running 3D analysis plug‐in to obtain lung volume. Tidal volume was calculated by subtracting EELV from EILV.

Adipose tissue in the thoracic area was delineated and quantified in a similar manner with lung volume quantification. To obtain an appropriate threshold, an intensity histogram of adipose tissue was extracted from a region of interest that contained only chest wall fat (Judex et al., 2010). After creating a VOI containing the dorsal half or ventral half of the chest, 3D analysis plug‐in was used to calculate adipose tissue amount in the dorsal and ventral side of the chest, respectively.

### 2.5 Upper airway measurements

Cross‐sectional areas (CSA) of the upper airway were measured with OsiriX 10.0 (Pixmeo) as detailed previously (Takahashi et al., 2020). CSA in the end‐expiration phase was measured at the following two locations: (1) at the end of the soft palate (caudal CSA; Brennick et al., 2009) and (2) 2 mm caudal to the edge of the hard palate (rostral CSA) at which the upper airway is closely surrounded by bones (Takahashi et al., 2020).

### 2.6 Statistical analysis

All statistical analyses were conducted using JMP 12.0 software (SAS Institute). Continuous variables are presented as mean and standard deviation. Mixed models were used to determine whether any change in EELV or upper airway dimensions was the result of the interaction between body weight and posture, followed by simple main effects analysis. Eta squared (*η* ^2^) represents the effect size of mixed models. Spearman's rank correlation coefficient (*ρ*) was used to evaluate the relationship between EELV and upper airway dimensions. The *p* value <0.05 was considered statistically significant for all analyses.

## 3 RESULTS

As compared to control mice (*n* = 5; age, 20 weeks; 32.92 ± 1.58 g), obese mice (*n* = 5; age, 20 weeks; 46.50 ± 2.77 g) had a significantly larger amount of chest wall fat (2756 ± 257 mm^3^ vs. 621 ± 109 mm^3^; *p* < 0.0001), which was especially notable in the dorsal side of the chest (obese mice: 1775 ± 212 mm^3^ vs. control mice: 356 ± 63 mm^3^; *p* < 0.0001).

**FIGURE 1:**
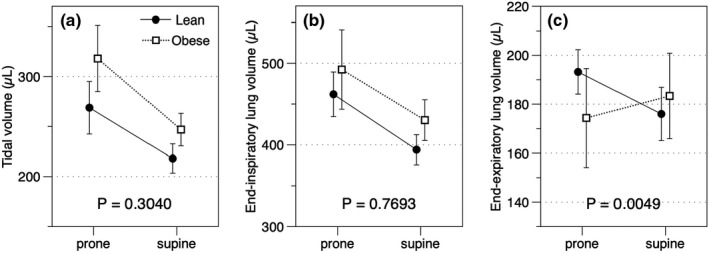
Interaction between the effects of weight and posture on lung volume. (a and b) No significant interaction between the effects of weight and posture on either tidal volume or end‐inspiratory lung volume (EILV) was observed (*p* = 0.3040 and 0.7693, respectively). Both lean and obese mice had a larger inspiratory lung volume and tidal volume during the prone position than the supine position (*p* < 0.0001 and 0.0002, respectively). (c) There was a statistically significant interaction between the effects of weight and posture on EELV (*p* = 0.0049). Simple main effects analysis revealed that EELV of control mice was significantly larger in the prone position than in the supine position (prone EELV = 193.22 ± 9.10 µl vs. supine EELV = 176.01 ± 10.91 µl; *p* = 0.0072). However, in contrast to control mice, obese mice had a lower EELV in the prone position although simple main effects analysis did not reach a statistically significance (prone EELV = 174.37 ± 20.23 µl vs. supine EELV = 183.39 ± 17.49 µl; *p* = 0.0981)

Both obese and lean mice showed a similar increase in tidal volume and EILV in the prone position (*p* = 0.3040 and 0.7693, respectively; Figure 1). However, a statistically significant interaction was observed between the effects of weight and posture on EELV (*p* = 0.0049, *η* ^2^ = 0.1041; Figure 1). Similar to tidal volume and EILV, EELV of control mice was significantly larger in the prone position than in the supine position (prone EELV = 193.22 ± 9.10 µl vs. supine EELV = 176.01 ± 10.91 µl; *p* = 0.0072). On the other hand, obese mice had a lower EELV in the prone position although simple main effects analysis did not reach a statistically significance (prone EELV = 174.37 ± 20.23 µl vs. supine EELV = 183.39 ± 17.49 µl; *p* = 0.0981).

**FIGURE 2:**
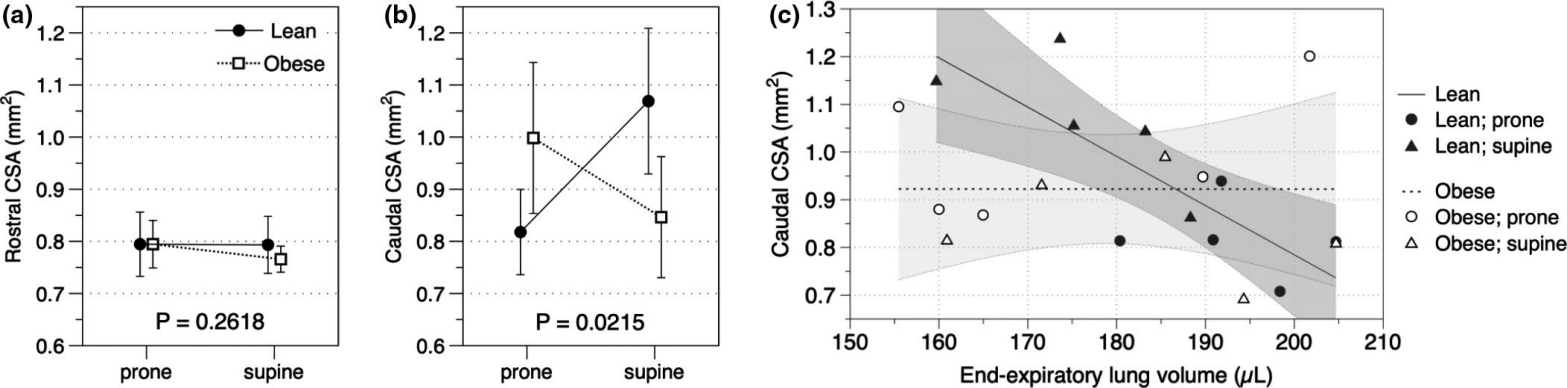
Interaction between the effects of weight and posture on upper airway dimensions. (a) Rostral CSAs were measured 2 mm caudal to the edge of the hard palate and did not differ between groups or body positions. (b) There was a statistically significant interaction between the effects of weight and posture on caudal CSA (*p* = 0.0215). Lean mice tended to have a larger caudal CSA in the supine position (*p* = 0.0947) while obese mice exhibited a significantly smaller caudal CSA in the supine position (*p* = 0.0472). (c) Lean mice had an inversed correlation between caudal CSA and EELV (Spearman's *ρ* = −0.818, *p* = 0.0038) while no correlation was observed in obese mice (Spearman's *ρ* = −0.1636, *p* = 0.6515). The lines indicate regression lines with shaded areas representing the corresponding 95% confidence intervals

Rostral CSAs were similar between groups and between postures (lean mice; prone CSA = 0.79 ± 0.06 mm^2^ vs. supine CSA = 0.79 ± 0.02 mm^2^; obese mice; prone CSA = 0.79 ± 0.05 mm^2^ vs. supine CSA = 0.77 ± 0.01 mm^2^; *p* = 0.2618; Figure 2A), while there was a statistically significant interaction between weight and posture on caudal CSA (*p* = 0.0215, *η* ^2^ = 0.6304; Figure 2B). Simple main effects analysis demonstrated that obese mice exhibited a significantly smaller caudal CSA in the supine position (prone CSA = 1.00 ± 0.14 mm^2^ vs. supine CSA = 0.85 ± 0.12 mm^2^; *p* = 0.0472). In lean mice, caudal CSA was inversely correlated with EELV (Spearman's *ρ* = −0.818, *p* = 0.0038). On the other hand, obese mice showed no correlation between EELV and caudal CSA (Spearman's *ρ* = −0.1636, *p* = 0.6515; Figure 2C), indicating that obese mice had a smaller upper airway (a reduced CSA) when EELV was low compared to lean mice.

## 4 DISCUSSION

To the best of our knowledge, the present study is the first experimental evidence indicating that diet‐induced obesity may lead to decreased EELV in mice in the prone position. We speculate that the mass loading effects of increased adipose tissue are likely the underlying mechanism of decreased EELV in high‐fat diet‐induced obese mice.

The small size of the mouse lung has posed a technical challenge for quantitative analysis of EELV in live mice (Irvin & Bates, 2003). Previously reported methods require mechanical ventilation with muscle paralysis and a tracheal cannula to measure EELV (Jánosi et al., 2006; Lundblad et al., 2002; Tankersley et al., 1998). In mice, unlike other mammals, inspiratory muscle tone is the determinant factor for EELV (Leith, 1983). Thus, the induction of muscle paralysis significantly alters lung compliance and is not technically suitable in evaluating changes in EELV under spontaneous breathing. Although we acknowledge that isoflurane use was a limitation possibly affecting muscle activity in the present study (Eastwood Peter et al., 2002), evaluating the upper airway with synchronization to spontaneous respiration without any invasive procedures is the major strength of our methods.

Body posture is an important factor independently influencing lung volume. The improvement in lung mechanical properties in the prone position is observed not only in humans but also in other mammals such as healthy pigs (Santini et al., 2015). The altered response observed in obese mice indicates that reduced chest wall compliance secondary to obesity diminished the advantageous effect of the prone position on lung mechanical properties. In contrast, both lean and obese mice showed similar changes in EILV and tidal volume with posture. These results suggest that static lung volumes such as EELV may be more vulnerable to obesity in mice. Another unique finding of the present study associated with posture is that obese mice showed a significantly smaller upper airway in the supine position than the prone position in the setting of the same head‐neck angle (prone, 127.79 ± 11.38 degrees vs. supine, 131.27 ± 5.00 degrees). One possible explanation for this finding is changes in the gravitational effect of soft tissues with posture. Indeed, it is known that obese mice have a larger tongue and soft palate due to fat deposition (Brennick et al., 2009), which may lead to the tongue falling backward in the supine position and compromise the upper airway especially in the setting of isoflurane use. In contrast, lean mice exhibited a larger caudal CSA in the supine position (Figure 2B). This may be explained by a compensation mechanism by upper airway dilator muscles to maintain upper airway patency when EELV is low given that the inverse correlation between caudal CSA and EELV was observed in lean mice (Figure 2C). Still, the etiology of upper airway narrowing and collapse is multifactorial, which is not fully characterized in this study. Furthermore, it remains unclear how our findings are relevant to sleep abnormalities in obese mice although we speculate that alterations of EELV would somewhat contribute to making the upper airway narrower or collapsible even in mice. Further research is required to clarify causal mechanisms linking EELV and body posture with airway calibers in mice. Additionally, the use of female mice and other strains will also be the future direction to clarify sex and strain differences in the relationship between EELV and airway calibers.

## 5 CONCLUSION

The effect of body weight on EELV changes depending on posture, and decreased EELV in the prone position appears to be the characteristic finding in obese mice. These alterations of EELV and upper airway calibers as a result of interaction effects between body weight and posture should be taken into consideration in future studies regarding upper airway collapse and lung mechanical properties using mice. We believe that our results will provide a better understanding of the interplay between EELV and the upper airway and facilitate the development of interventions directed at the pathophysiology associated with EELV.

## CONFLICT OF INTEREST

No conflict of interest, financial or otherwise, are declared by the authors.

## AUTHOR CONTRIBUTIONS

T.T. designed the study, acquired and analyzed the data, and wrote the manuscript. N.S. and S.N. designed the study and revised the manuscript.

## Data Availability

The data that support the findings of this study are available from the corresponding author upon reasonable request.
